# Peptide Inhibitor of Complement C1 Inhibits the Peroxidase Activity of Hemoglobin and Myoglobin

**DOI:** 10.1155/2017/9454583

**Published:** 2017-09-10

**Authors:** Pamela S. Hair, Kenji M. Cunnion, Neel K. Krishna

**Affiliations:** ^1^Department of Pediatrics, Eastern Virginia Medical School, 700 West Olney Road, Norfolk, VA 23507, USA; ^2^Department of Microbiology and Molecular Cell Biology, Eastern Virginia Medical School, 700 West Olney Road, Norfolk, VA 23507, USA; ^3^Children's Specialty Group, 811 Redgate Avenue, Norfolk, VA 23507, USA; ^4^Children's Hospital of The King's Daughters, 601 Children's Lane, Norfolk, VA 23507, USA

## Abstract

Hemoglobin is the natural carrier of oxygen in red blood cells (RBCs). While intracellular hemoglobin provides life-sustaining oxygen transport, extracellular free hemoglobin displays toxicity due to inherent peroxidase activity generating reactive oxygen species that subsequently react with the hemoglobin molecule to produce toxic heme degradation products resulting in free radicals, oxidative stress damage, and lipid peroxidation. We have recently demonstrated that Peptide Inhibitor of Complement C1 (PIC1) inhibits peroxidase activity of the heme-based enzyme myeloperoxidase. To elucidate whether PIC1 could inhibit peroxidase activity of hemoglobin, we evaluated the consequence of PIC1 on RBC lysates, methemoglobin, and myoglobin using tetramethylbenzidine (TMB) as an oxidation target. PIC1 reversibly and dose-dependently prevented TMB oxidation to tetramethylbenzidine diimine by RBC lysates, methemoglobin, and myoglobin, having comparable activity to the inhibitor 4-aminobenzoic acid hydrazide. PIC1 inhibited TMB oxidation of RBC lysates similar to L-cysteine suggesting that the two cysteine residues contained in PIC1 may mediate peroxidase activity. PIC1 also inhibited heme destruction by NaOCl for RBC lysates, hemoglobin, and myoglobin as assayed by preservation of the Soret absorbance peak in the presence of NaOCl and reduction in free iron release. In conclusion, PIC1 inhibits peroxidase activity of hemoglobin and myoglobin likely via an antioxidant mechanism.

## 1. Introduction

The hemoglobin (Hb) molecule is a protein tetramer composed of two alpha and two beta chains each of which coordinates an iron-containing heme prosthetic group [1]. Hb constitutes up to 92% of the dry content of the red blood cell (RBC) and plays the essential role of delivering oxygen from the lung to the peripheral tissue for cellular uptake reciprocally removing carbon dioxide from the tissue to the lung [1]. While Hb provides life-sustaining oxygen delivery, the Hb molecule is inherently toxic due to its well-characterized redox activity leading to autoxidation of the ferrousHb (Fe^2+^) oxygen containing Hb (oxyHb) into nonfunctional ferricHb (Fe^3+^) also known as methemoglobin (metHb) and reactive oxygen species (ROS) generation of superoxide ion (O_2_^•−^) [2]. Within the confines of the RBC, autoxidation is minimized by the metHb reductase system; however free metHb can undergo a further peroxidase reaction with O_2_^•−^ to generate ferrylHb (Fe^4+^) and the ROS hydrogen peroxide (H_2_O_2_) that in turn attacks Hb to produce protein radicals, irreversibly damaging the Hb molecule leading to heme degradation and release of free iron [2]. In addition to O_2_^•−^ and H_2_O_2_, the ROS hypochlorous acid (HOCl), generated by myeloperoxidase (MPO) present in neutrophil and macrophage granules, has been demonstrated to bind to the heme moiety of Hb causing heme destruction, protein modification, and protein aggregation as well as formation of additional peroxidase-like activity [3, 4].

Hb peroxidase activity and its degradation are extremely toxic to organs and tissues, having serious pathological ramifications [5, 6]. In addition to generation of ROS from free Hb, iron released from heme degradation can result in the generation of additional ROS resulting in cellular damage. Further reaction of free iron with H_2_O_2_ forms a potent oxidant that is damaging to lipids, nucleic acids, and amino acids. Finally, Hb protein radical formation can result in crosslinking of heme and amino acids [2]. Currently, there are no pharmacological agents available on the market to control pathological extracellular Hb-mediated oxidative reactions.

Our laboratory has characterized a family of peptides know as Peptide Inhibitor of Complement C1 (PIC1) that inhibit the classical pathway of complement by binding the initiator molecule C1q and inhibiting activation of the cognate serine protease tetramer C1s-C1r-C1r-C1s that is normally associated with C1q [7, 8]. We have recently demonstrated that the lead, pegylated PIC1 compound, PA-dPEG24 (amino acid sequence IALILEPICCQERAA containing a 24 units PEG moiety attached to the C terminus, total molecular weight 2,771 Da), can inhibit MPO activity in cystic fibrosis sputum, as well as from neutrophils and purified MPO [9]. To ascertain if PIC1 had any inhibitory activity on Hb, which shares with MPO a heme-based mechanism of action, we analyzed the effect of PIC1 on the peroxidase activity of Hb from RBCs. PIC1 was able to inhibit this activity by Hb, as well as purified metHb and myoglobin and also protect its destruction by sodium hypochlorite (NaOCl), suggesting that PIC1 possesses a novel antioxidant activity that may be of therapeutic benefit.

## 2. Materials and Methods

### 2.1. Ethics Statement

Human blood was obtained from four healthy volunteers for the generation of RBC lysates used in these studies, per the Eastern Virginia Medical School IRB approved protocol 02-06-EX-0216. The study participants provided written informed consent. Human blood was collected for these experiments from May 2016 through November 2016.

### 2.2. Materials

PIC1 (IALILEPICCQERAA-dPEG24) was manufactured by PolyPeptide Group (San Diego, CA) to ≥95% purity as verified by HPLC and mass spectrometry analysis. Lyophilized PIC1 was solubilized in normal saline with 0.01 M Na_2_HPO_4_ buffer to 15 mM. dPEG24 was purchased from PolyPeptide Group and dissolved in the same buffer. Pure human methemoglobin (metHb), myoglobin from equine skeletal muscle, and L-cysteine were purchased from Sigma (St. Louis, MO). 3,3′,5,5′-Tetramethylbenzidine (TMB) as a solution containing H_2_O_2_ was purchased from Thermo Fisher Scientific (Waltham, MA). 4-Aminobenzoic acid hydrazide (ABAH), O-dianisidine, and NaOCl were purchased from Sigma-Aldrich (St. Louis, MO). 2,2′-Azino-bis(3-ethylbenzothiazoline-6-sulphonic acid) (ABTS) solution was purchased from Thermo Fisher Scientific (Waltham, MA).

### 2.3. Preparation of Human RBC Lysate

RBC lysates were prepared from the blood of healthy donors as previously described [10]. Cells were washed twice with GVBS^−−^ (veronal-buffered saline, 0.1% gelatin, and 0.01 M EDTA) and then incubated at 37°C to allow for spontaneous lysis. The cells were then washed twice again with GVBS^++^ (veronal-buffered saline, 0.15 mM CaCl_2_, and 1.0 mM MgCl_2_) and resuspended in a volume which set the concentration to 1 × 10^9^ cells/ml (absorbance reading of lysed RBCs at 541 nm of 0.210 = 1 × 10^9^ cells/ml). An aliquot of these cells was combined with an equal aliquot of water at room temperature and spun at 21,000 ×g for 5 minutes to separate the released hemoglobin from the cell debris.

### 2.4. Measurement of Peroxidase Inhibition by PIC1

Inhibition of peroxidase activity was assessed by combining the RBC lysate (reported in cellular equivalents/ml), metHb (0.5 mg/ml), or myoglobin (0.5 mg/ml) with increasing concentrations of PIC1, with a constant final volume, for 5 minutes before adding TMB as described below. Additionally, increasing amounts of RBC lysates were incubated with increasing amounts of PIC1, with a constant final volume, for 5 minutes before adding TMB. As a positive control for inhibition of peroxidase activity, increasing amounts of ABAH or L-cysteine were included in select experiments. After the 5-minute incubation, samples were combined with 100 *μ*l of TMB in a nontreated 96-well plate for 15 seconds followed by 100 *μ*l of 2.5 N H_2_SO_4_ (stop buffer) and absorbance was read at 450 nm in a Synergy HT BioTek plate reader [10]. For assays using ABTS, 150 *μ*l of ABTS solution was added to each sample and incubated for 15 seconds before adding 100 *μ*l of 1% SDS to stop the reaction [11]. Absorbance was read at 405 nm. O-Dianisidine working solution was prepared by making a 0.166 mg/ml solution in 50 mM phosphate-citrate buffer, pH 5.0 with a final of 0.006% H_2_O_2_ added immediately before use [12]. 100 *μ*l of the prepared O-dianisidine solution was added to each sample and incubated for 2 minutes before adding 100 *μ*l of 2.5 N H_2_SO_4_ to stop the reaction. Absorbance was read at 405 nm.

To assess if PIC1 could inhibit peroxidase activity of Hb released from hemolyzed RBCs in human serum, type O sera at 15% in GVBS++ buffer was mixed with type AB RBCs at titrating concentrations (1 : 2 dilutions of 5 × 10^7^ RBCs) followed by incubation for 1 hour at 37°C to allow for RBC hemolysis via antibody mediated complement lysis [13]. Samples were then centrifuged and supernatant containing the released Hb collected. PIC1 at 1 mM final concentration was subsequently combined with the sample supernatants and allowed to incubate for 5 minutes before adding TMB and H_2_SO_4_ as above to evaluate peroxidase activity from the cell-free Hb.

### 2.5. Reversal of Peroxidase Activity by PIC1

RBC lysate (2.5 × 10^8^ cells/ml equivalent), 2 mg/ml metHb, or 2 mg/ml myoglobin was coated onto an Immunlon-2, 96-well plate in a carbonate coating buffer (0.16 M Na_2_CO_3_, 0.34 NaHCO_3_, pH 9.6) overnight at 4°C. The plate was then washed; TMB was added and allowed to incubate for 1 hour. The oxidized TMB was transferred to a fresh well containing either water or 2 mM PIC1. The reaction was stopped after 1 min with 2.5 M H_2_SO_4_. Absorbance was read at 450 nm.

### 2.6. Free Iron Assay

Iron release from the RBC lysate, metHb, or myoglobin was measured utilizing a ferrozine assay [14]. After the absorbance spectra were recorded, 100 *μ*l of ascorbic acid (100 mM) was added to each sample and allowed to incubate for 5 minutes. Then 50 *μ*l of ammonium acetate (16%) and 50 *μ*l of ferrozine (16 mM) were added to the samples and mixed. After incubating for 5 minutes, absorbance was measured at 562 nm. A standard curve was prepared using ammonium Fe(III) sulfate and a linear regression was used to calculate the free iron concentration.

### 2.7. Spectral Analysis of RBC Lysate and Pure metHb

Absorbance spectra experiments were performed with 1.25 × 10^7^ cells/ml RBC lysate, 0.2 mg/ml metHb, or 0.2 mg/ml myoglobin followed by various concentrations of PIC1, ABAH, and NaOCl (at a 1 : 1,000 dilution), in a nontreated 96-well plate. As each component was added to the RBC lysate, metHb, or myoglobin, it was allowed to incubate for 5 minutes. Absorbance values were recorded from 300 to 550 nm [14].

### 2.8. Statistical Analysis

Quantitative data were analyzed determining means, standard error (SEM), and Student's *t*-test using Excel (Microsoft, CA). *P* values ≤ 0.05 were considered statistically significant.

## 3. Results

### 3.1. PIC1 Inhibits Peroxidase Activity of Hb in RBC Lysates

**Figure 1:**
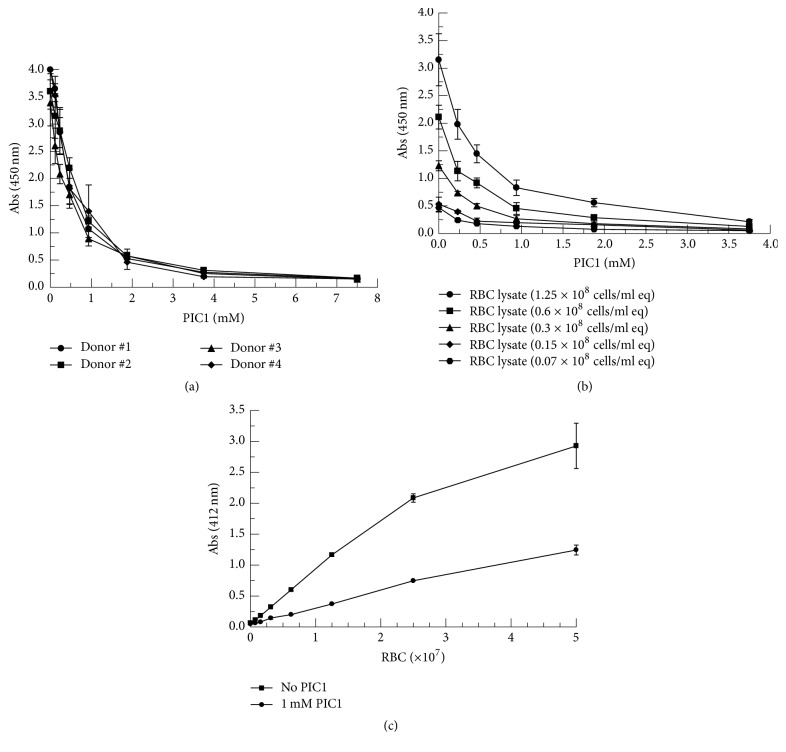
PIC1 inhibits peroxidase activity of Hb in RBC lysates. (a) RBC lysates (1.25 × 10^8^ cells/ml equivalents (eq)) from four donors were incubated with increasing concentrations of PIC1. After a 5-minute incubation, samples were combined with TMB for 15 seconds and absorbance was read at 450 nm. Data are means of three independent experiments ± SEM. (b) Dose-response inhibition of RBC lysates from one donor at various concentrations by PIC1. Data are means of four independent experiments ± SEM. (c) Increasing amounts of human AB RBCs were added to human O serum and cells allowed to lyse. PIC1 was then added to the serum aliquots and after a 5-minute incubation, samples were combined with TMB for 15 seconds and absorbance was read at 412 nm. Data are means of three independent experiments ± SEM.

In an attempt to ascertain whether PIC1 would have any effect on the peroxidase activity of extracellular Hb, RBC lysates were prepared from four donors and incubated with increasing amounts of PIC1 followed by TMB as the target for oxidation. Addition of PIC1 led to a dose-dependent inhibition of the TMB oxidation product 3,3′,5,5′-tetramethylbenzidine diimine (Figure 1(a)) demonstrating a 24.8-fold reduction in peroxidase activity for 7.5 mM PIC1 compared with no PIC1 (*P* < 0.001). In order to further evaluate PIC1 inhibition of peroxidase activity over a range of RBC lysate concentrations, a more extensive dose-response experiment was conducted with RBC lysates from one donor. Concentration-dependent PIC1 inhibition of RBC lysate peroxidase activity was demonstrated for all RBC lysate concentrations (Figure 1(b)). PIC1 at 3.75 mM inhibited peroxidase activity 14.3-fold compared with no PIC1 at 1.25 × 10^8^ RBC/ml equivalents (*P* = 0.007).

In an attempt to assess the peroxidase activity of PIC1 in a scenario mimicking intravascular hemolysis, the following experiment was performed. Increasing amounts of human AB RBCs were incubated in the presence of O serum which contains antibodies to the A and B glycoproteins resulting in complement-mediated lysis of the RBCs [13]. When PIC1 was added to the serum sample containing the lysed RBCs followed by TMB, PIC1 was found to inhibit peroxidase activity of the released Hb compared to the no PIC1 control 2.4-fold at 5.0 × 10^7^ RBCs (Figure 1(c)). These results suggest that PIC1 can inhibit peroxidase activity of Hb from RBCs lysed in a complement-mediated reaction in human serum.

### 3.2. PIC1 Reversibly Inhibits Peroxidase Activity of RBC Lysates, metHb, and Myoglobin in a Comparable Manner to ABAH

**Figure 2:**
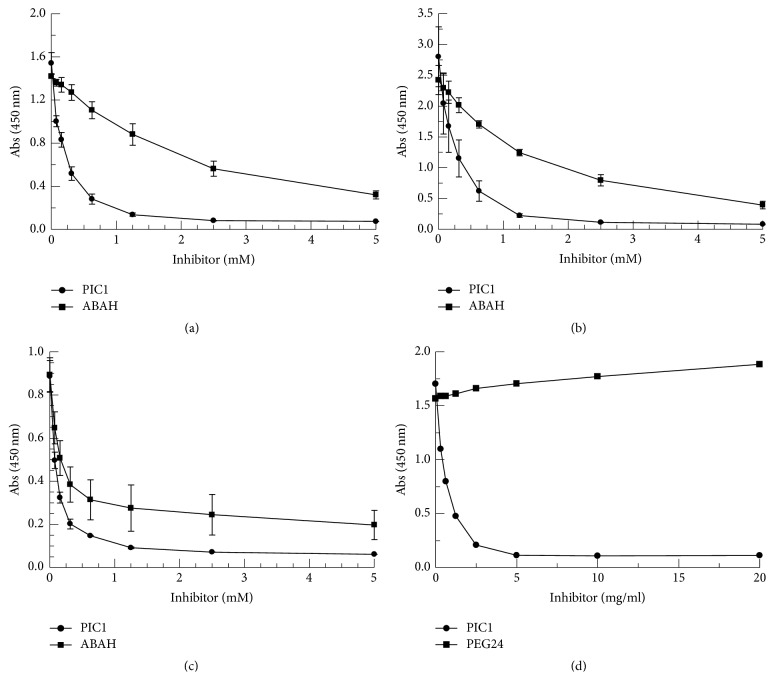
Comparison of PIC1 and ABAH inhibition of peroxidase activity of metHb, Hb from RBC lysates, and myoglobin. (a) 0.5 mg/ml metHb, (b) RBC lysates (1.25 × 10^8^ cells/ml equivalents), or (c) 0.5 mg/ml myoglobin were incubated with increasing concentrations of PIC1 and ABAH. Samples were then combined with TMB and absorbance was read at 450 nm. Data are means of three independent experiments ± SEM. (d) 0.5 mg/ml metHb was incubated with increasing concentrations of PIC1 and PEG24. Samples were then combined with TMB and absorbance was read at 450 nm.

To ascertain that the PIC1-mediated inhibition of peroxidase activity demonstrated for the RBC lysate was indeed due to Hb alone, PIC1 was incubated with pure metHb. As a positive control for peroxidase inhibition, 4-aminobenzoic acid hydrazide (ABAH) was utilized. ABAH is an inhibitor that specifically and irreversibly inhibits the peroxidase activity of MPO, a heme containing enzyme produced by neutrophils and macrophages [15, 16]. On a molar basis, PIC1 demonstrated a similar dose-dependent inhibition of the peroxidase activity of metHb as seen with ABAH (Figure 2(a)). At 1.25 mM, PIC1 exhibited a 6.4-fold (*P* = 0.004) improvement in metHb inhibition compared with ABAH. As expected, the PIC1 inhibition of metHb peroxidase activity was very similar to that of the RBC lysate tested in parallel which demonstrated superior activity to ABAH (Figure 2(b)). At 1.25 mM, PIC1 showed a 5.6-fold (*P* = 0.009) improvement in Hb inhibition compared with ABAH. In order to determine if the peroxidase inhibitory activity of PIC1 was active against another toxic heme-bearing enzyme, the TMB assay was performed with pure myoglobin. PIC1 dose-dependently inhibited the peroxidase activity of myoglobin by 14.3-fold (*P* = 0.006) at 5 mM compared with no PIC1 (Figure 2(c)). PIC1 inhibition of myoglobin peroxidase activity was not statistically different from ABAH. Polyethylene glycol (PEG) has been previously reported to have antioxidant capacity [17]. To ascertain whether the observed peroxidase inhibiting activity of PIC1 was due to its C terminal PEG tail which is composed of 24 polyethylene glycol units (PEG24), PIC1 or purified PEG24 was incubated with metHb. While PIC1 dose-dependently inhibited peroxidase activity, PEG24 had no effect (Figure 2(d)).

**Figure 3:**
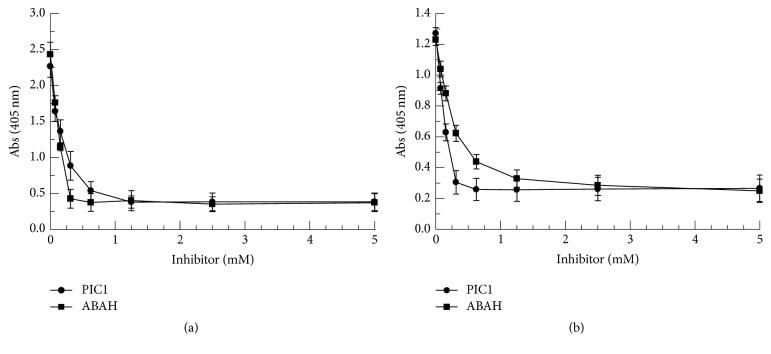
Comparison of PIC1 and ABAH inhibition of peroxidase activity of Hb from RBC lysates using ABTS and O-dianisidine as substrates. RBC lysates (1.25 × 10^8^ cells/ml equivalents) were incubated with increasing concentrations of PIC1 or ABAH. Samples were then combined with (a) ABTS or (b) O-dianisidine and absorbance was read at 405 nm. Data are means of three independent experiments ± SEM.

To rule out that PIC1 inhibition of extracellular Hb-mediated TMB oxidation in this assay was specific to the substrate used, the reagents ABTS and O-dianisidine were utilized in place of TMB. Both PIC1 and ABAH showed dose-dependent inhibition of free Hb peroxidase activity for ABTS (Figure 3(a)) and O-dianisidine (Figure 3(b)) [11, 12]. Additionally, PIC1 incubated in the presence of TMB alone did not result in a change in absorbance at 450 nm, confirming that PIC1 does not directly reduce the reporter probe. Collectively, these data suggest that PIC1 is able to broadly inhibit the peroxidase activity of the heme-bearing enzymes Hb and myoglobin.

**Figure 4:**
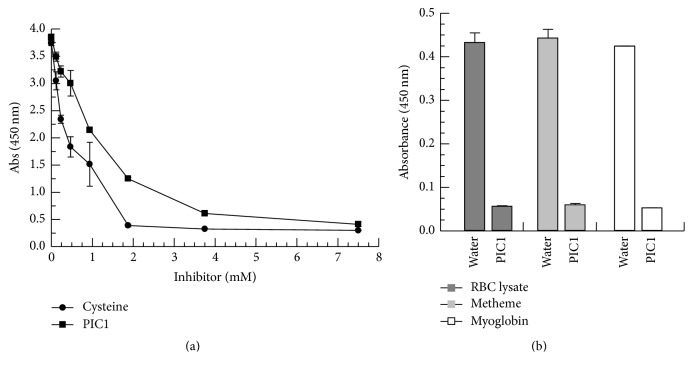
PIC1 and cysteine show similar inhibition of TMB oxidation that is reversible. (a) RBC lysates were incubated with increasing concentrations of PIC1 and L-cysteine. Samples were then combined with TMB and absorbance was read at 450 nm. Data are means of three independent experiments ± SEM. (b) RBC lysate (2.5 × 10^8^ cells/ml equivalents), 2 mg/ml metHb, or 2 mg/ml myoglobin was coated onto an Immunlon-2, 96-well plate, and incubated overnight. The plate was then washed and TMB added for 1 hour to promote oxidation. The oxidized material was then added to a fresh well containing either water or 2 mM PIC1. Samples were then measured by absorbance at 450 nm. Data are means of three independent experiments ± SEM.

PIC1 contains vicinal cysteine residues at positions 9 and 10 of the peptide. It is well established that the thiol group of cysteine residues are efficient peroxidase inhibitors with L-cysteine demonstrating significant antioxidant activity [18]. In order to evaluate the level of PIC1 peroxidase inhibition compared with the cysteine, equivalent molar amounts of L-cysteine and PIC1 were added to RBC lysates and analyzed in the TMB assay. PIC1 and L-cysteine both demonstrated a dose-dependent and similar inhibition of peroxidase activity (Figure 4(a)). This data suggests that the vicinal cysteines of PIC1 could mediate the peroxidase inhibitory activity of the peptide.

In an attempt to ascertain whether PIC1 could be inhibiting peroxidase activity via an antioxidant effect (i.e., determine whether oxidation can be reversed) the following experiment was conducted. RBC lysate, metHb, or myoglobin was coated onto a 96-well plate and incubated overnight. The plate was then washed and TMB added for 1 hour to allow the substrate to oxidize. Each solution was then transferred to a fresh well which contained either water or PIC1. PIC1 was able to reverse the oxidation state of TMB for the RBC lysate, metHb, and myoglobin as measured by absorbance at 450 nm with a 7.2–8.4-fold (*P* ≤ 0.003) reduction in oxidized TMB (3,3′,5,5′-tetramethylbenzidine diimine) for each of the proteins incubated with PIC1 compared to the water only control (Figure 4(b)).

### 3.3. PIC1 Prevents Hb and Myoglobin Destruction and Iron Release Mediated by NaOCl

**Figure 5:**
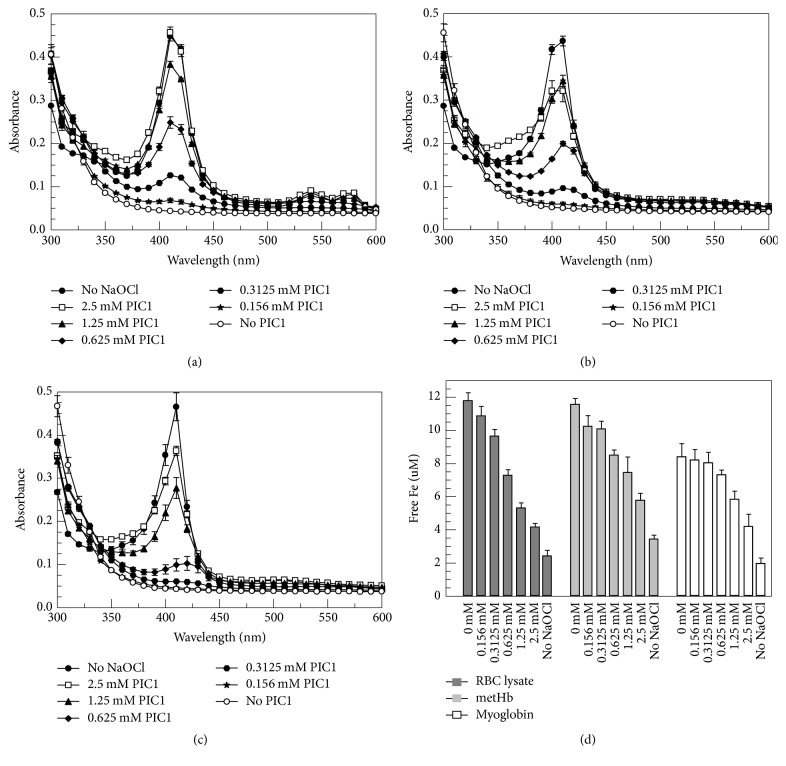
PIC1 prevents Hb and myoglobin destruction and iron release mediated by NaOCl. (a) RBC lysates (1.25 × 10^7^ cells/ml equivalents), (b) 0.2 mg/ml metHb, and (c) 0.2 mg/ml myoglobin were added to increasing concentrations of PIC1 followed by a 1 : 1,000 dilution of NaOCl. Samples were then incubated for 5 minutes and spectral absorbance readings were recorded from 300 to 550 nm. (d) Free iron (Fe) release from each sample was determined by the ferrozine assay. Data are means of three independent experiments ± SEM.

Hypochlorous acid (HOCl) produced by MPO present in phagocyte granules has been shown to mediate heme destruction and free iron release [3, 4]. HOCl induced changes to the heme ring and iron atom of Hb may be evaluated by measuring spectral absorption to monitor the Soret peak which is characteristic of porphyrin containing compounds such as the heme moiety of Hb and myoglobin [4]. To ascertain if PIC1 was able to protect the heme group from hypochlorite attack, RBC lysates, metHb, and myoglobin were incubated with increasing concentrations of PIC1 followed by NaOCl and spectral analysis performed. Compared to no PIC1 treatment (NaOCl alone), RBC lysates incubated with increasing amounts of PIC1 showed a dose-dependent protection of the Soret peak (Figure 5(a)). The same result was obtained for pure metHb (Figure 5(b)) and myoglobin (Figure 5(c)). To assess whether PIC1 also prevented the release of free iron from the heme group of Hb and myoglobin, the ferrozine assay was performed on these samples. PIC1 dose-dependently inhibited free iron release for RBC lysates, metHb, and myoglobin (*P* ≤ 0.003 for 2.5 mM PIC1 versus no PIC1) (Figure 5(d)) consistent with the spectral absorbance data showing protection of the heme group.

**Figure 6:**
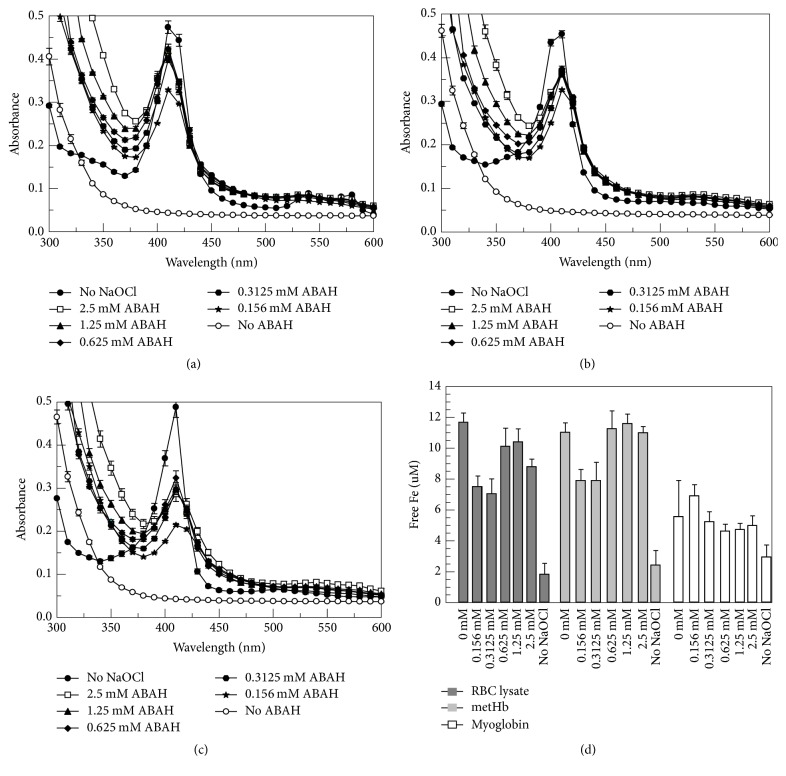
ABAH prevents Hb and myoglobin destruction but does not prevent iron release mediated by NaOCl. (a) RBC lysates (1.25 × 10^7^ cells/ml equivalents), (b) 0.2 mg/ml metHb, and (c) 0.2 mg/ml myoglobin were added to increasing concentrations of ABAH followed by a 1 : 1,000 dilution of NaOCl. Samples were then incubated for 5 minutes and spectral absorbance readings were recorded from 300 to 550 nm. (d) Free iron (Fe) release from each sample was determined by the ferrozine assay. Data are means of three independent experiments ± SEM.

Given the similar levels of peroxidase inhibition by ABAH and PIC1 shown in the TMB assay (Figures 2(a)–2(c)), we next analyzed whether ABAH produced the same protective effect against NaOCl as seen with PIC1. ABAH showed protection of the Soret peak of Hb from the RBC lysate (Figure 6(a)) as well as metHb (Figure 6(b)) and myoglobin (Figure 6(c)). Interestingly, in all three cases, ABAH did not demonstrate a dose-dependent inhibition as seen with PIC1; each concentration of ABAH protected the Soret peak of Hb to a similar degree. Additionally, while various concentrations of ABAH prevented flattening of the Soret peak in the presence of NaOCl, this inhibitor was unable to block release of free iron in a dose-dependent fashion for the RBC lysate, metHb, and myoglobin (Figure 6(d)). These data suggest that PIC1 protection of these enzymes from NaOCl may occur through a different mechanism of action than what is observed for ABAH.

## 4. Discussion

Heme is an essential component of proteins such as Hb and myoglobin where it functions in life-sustaining oxygen transport and storage, respectively. However, severe hemolysis leading to release of free Hb plays a significant role in pathological conditions such as sickle cell disease, ischemia-reperfusion injury, malaria, hemolytic anemia, blood transfusion, and renal failure [5, 6]. Rhabdomyolysis results in the release of toxic myoglobin [19]. While haptoglobin can bind some free Hb and heme oxygenase (HO) serves as a heme detoxification system, in the situation of massive free heme accumulation these systems are overwhelmed thus allowing free heme to exert its toxic effects [5]. Free heme results in the formation of ROS that lead to oxidative stress resulting in lipid peroxidation and DNA damage as well as protein aggregation and damage [2]. In addition, free heme possesses proinflammatory properties that lead to neutrophil activation, which in turn induces chemotaxis, cytokine production, and additional ROS as well as HOCl generation [5]. Finally, free heme can bind RBCs and through a number of different mechanisms cause additional hemolysis [5]. Given the highly lethal properties of free heme, development of an inhibitor to detoxify the oxidative properties of free heme in acute hemolysis and rhabdomyolysis remains major unmet medical needs.

The experiments shown here demonstrate that PIC1 can dose-dependently inhibit the peroxidase activity of heme from RBC lysates as well as pure metHb and myoglobin for three different substrates: TMB, ABTS, and O-dianisidine. The level of inhibition was comparable to or better than that of the MPO inhibitor ABAH. Furthermore, PIC1 was able to reverse the oxidation of TMB for RBC lysates, metHb, and myoglobin. PIC1 was found to dose-dependently inhibit NaOCl-mediated destruction of heme and prevent the release of free iron as shown by preservation of the Soret peak in the spectral assay and the ferrozine assay, respectively. Interestingly, while ABAH was shown to inhibit Soret peak flattening, the inhibition was not dose-dependent and did not prevent free iron release, suggesting a different mechanism of Hb protection from NaOCl by PIC1 versus ABAH. The inhibition of Hb and myoglobin peroxidase activity are consistent with our recently published results demonstrating that PIC1 inhibits peroxidase activity of MPO [9]. Together these results suggest that PIC1 can broadly inhibit diverse heme-bearing enzymes.

PIC1 derivative PA-dPEG24 contains two vicinal cysteine residues at positions 9 and 10. It is possible that the observed inhibition of peroxidase activity for metHb and myoglobin is mediated by the reduction of higher oxidation states of these proteins by these cysteine residues. Oxidation of the cysteine residues would be predicted to either result in intermolecular disulphide bonds between the cysteine residues of two PIC1 molecules or possibly intramolecular cystine formation. PIC1 was found to dose-dependently inhibit peroxidase activity of RBC lysates in a similar manner to L-cysteine (Figure 4(a)) suggesting that these residues may mediate the peroxidase inhibitory activity of PIC1. The possibility that the cysteine residues of PIC1 facilitate peroxidase inhibition of these heme-bearing enzymes also may explain the observed differences in the level of peroxidase inhibition of ABAH versus PIC1 (Figure 2) as well as Hb and myoglobin destruction and iron release mediated by NaOCl (Figures 5 and 6). Thiol groups have been documented to possess different reactivity to free radicals and heme high valent complexes compared to hydrazides like ABAH [20, 21]. While the cysteine residues of PIC1 are essential for the prevention of peroxidase activity, they also are critical for the complement inhibitory activity of this peptide as substitution of either of the two cysteine residues in earlier derivatives of PIC1 inhibited complement activity [22]. Experiments are currently underway to determine if these cysteine amino acids exist as free thiols in solution and if the mechanism by which PIC1 inhibits peroxidase activity involves oxidation of one or both of these residues.

Free heme is exquisitely toxic to the kidney where it can lead to renal failure through a number of mechanisms including oxidative damage and heme deposition in the renal tubules [5]. We have recently developed a rat model of intravascular hemolytic transfusion reaction in which Wistar rats are transfused with incompatible RBCs [10]. The transfused cells lyse, releasing free Hb which results in acute kidney disease at 6 hours after transfusion as assessed by increased kidney weight and tissue damage by histopathology. Animals prophylactically treated with PIC1 showed protection of the kidneys compared to animals receiving either saline only or intravenous immunoglobulin (IVIG) [23]. We speculate that PIC1 may be mediating kidney protection in this animal model through two independent, anti-inflammatory actions: (1) inhibition of complement-mediated RBC lysis preventing free Hb release and (2) inhibition of the peroxidase activity of Hb. Future experiments to explore these dual functions of the PIC1 molecule in this animal model are planned. As there are currently no marketed pharmacological inhibitors for the prevention of extracellular heme-mediated peroxidation, PIC1 may have utility as a therapeutic agent for diseases involving intravascular hemolysis and rhabdomyolysis.

## 5. Conclusions

Free Hb from lysed RBCs and myoglobin released from damaged muscle cells are inherently toxic molecules in the blood stream due to their intrinsic peroxidase activity and have been demonstrated to contribute to pathogenesis in hemolytic diseases and rhabdomyolysis, respectively. Currently there are no inhibitors of peroxidase activity in the marketplace to address these unmet medical needs. Peptide Inhibitor of Complement C1 (PIC1) significantly inhibits peroxidase activity of RBC lysates and purified Hb as well as myoglobin. Based on these findings, future experiments to test PIC1-mediated inhibition of peroxidase activity in preclinical animal models are currently planned.
